# Antimicrobial effects of XF drugs against *Candida albicans* and its biofilms

**DOI:** 10.3389/ffunb.2023.1225647

**Published:** 2023-08-22

**Authors:** E. L. Board-Davies, W. Rhys-Williams, D. Hynes, W. G. Love, D. W. Williams

**Affiliations:** ^1^ School of Dentistry, Cardiff University, Cardiff, United Kingdom; ^2^ Destiny Pharma plc, Brighton, United Kingdom

**Keywords:** *Candida albicans*, biofilms, antifungals, XF drugs, infection

## Abstract

Compared with antibiotics for treating bacterial infections, there are a limited number of antifungal agents. This is due to several factors, including the difficulties of identifying suitable antifungals that target the fungal cell without damaging host cells, and the reduced rates of diagnosis of fungal infections compared with those caused by bacteria. The problem of treating fungal infections is exacerbated by an increasing incidence of antifungal resistance among human fungal pathogens. Three XF drugs (XF-73, XF-70, and DPD-207) have previously displayed innate bactericidal effects and a low propensity for microbial resistance, with XF-73 and XF-70 having a second, light-activated mechanism of action [known as photodynamic therapy (PDT)]. In an effort to expand the repertoire of antifungal agents, this research assessed the *in vitro* activity of XF drugs via both mechanisms of action against six strains of the fungal pathogen *Candida albicans* in both planktonic and biofilm cultures. In addition, this research examined the effects of XF drug treatment on biofilms of *C. albicans* in a reconstituted human oral epithelium model. All *C*. *albicans* strains tested were susceptible to XF-73 and XF-70, with minimum inhibitory concentrations (MICs) between 0.25 µg/mL and 2 µg/mL; DPD-207 was less potent, with MICs between 4 µg/mL and 16 µg/mL, and light activation did not enhance these MICs. Complete biofilm eradication was not reported at the tested XF drug concentrations. However, live and dead staining of *C. albicans* cells in biofilms after XF drug treatment demonstrated that XF-73 and XF-70 were active against most *Candida* biofilms tested from 64 µg/mL; again, light activation did not enhance anti-biofilm activity. *Candida* biofilms were more resistant to DPD-207, with fungicidal effects occurring from 256 µg/mL. XF-73 and XF-70 reduced penetration of *C. albicans* biofilm into reconstituted human oral epithelium (RHOE) and resulted in less damage (as determined by reduced lactate dehydrogenase release) than untreated biofilms. Overall, the results highlight the potential of XF drugs as new drugs for the management of topical infections caused by *C. albicans*. Further studies are warranted on the development of XF drugs as antifungals, particularly for XF-73 and XF-70.

## Introduction

The fungal genus of *Candida* contains over 150 species, with several species associated with human infection (candidosis). The most prevalent cause of candidosis is *Candida albicans*, which typically colonises humans as a harmless commensal but can cause infections when conditions allow (Patel, 2022). The majority of these infections are topical, affecting the oral and vaginal mucosa, but in severely immunocompromised individuals, serious systemic infection can arise (Brown et al., 2012). Globally, *C. albicans* is responsible for > 150 million mucosal infections, with an estimated 200,000 deaths per annum occurring due to invasive and disseminated disease in susceptible populations, (Richardson, 2022).

Four clinical presentations of primary oral candidosis are recognised: chronic erythematous candidosis, chronic hyperplastic candidosis (Lorenzo-Pouso et al., 2022), acute erythematous candidosis (Gonsalves et al., 2008), and acute and chronic pseudomembranous candidosis (Baumgardner, 2019). In addition, there are oral conditions associated with secondary *Candida* infection, including angular cheilitis, central papillary atrophy (median rhomboid glossitis), and lichen planus. Host-associated risk factors for oral candidosis include nutritional deficiencies, hormonal imbalances, receipt of broad-spectrum antibiotics or immunosuppressive therapies, poor oral hygiene, and inappropriate denture care and use (Pankhurst, 2013). To instigate infection, several putative *C. albicans* virulence factors are recognised, including the ability to generate tissue-invading filamentous forms (hyphae and pseudohyphae) and production of hydrolytic enzymes, such as secreted aspartyl proteinases (SAPs) and phospholipases (PLs) (Bu et al., 2022). There is also the prerequisite for *Candida* to be able to adhere to oral surfaces and form biofilms (Rautemaa and Ramage, 2011).

Biofilms are defined as microbial communities that adhere to biotic and abiotic surfaces, with the microbial cells being embedded in a self-produced extracellular polymeric substance (EPS) (Mishra et al., 2023). The biofilm cells are protected from removal from the surface largely because the EPS provides adhesive and cohesive forces of attachment and impedes access by host immune factors and administered antimicrobials. Importantly, biofilm cells can be up to 1,000 times more resistant to antimicrobials than their planktonic or free-living counterparts (Rogers et al., 2010). The successful treatment of fungal biofilms is hindered by their inherent barrier resistance, the relatively limited numbers of effective antifungal drugs compared with the numbers of antibiotics, and the emergence of antifungal drug resistance (Gong et al., 2023).

The majority of currently licensed antifungals belong to one of four classes (i.e., azoles, polyenes, echinocandins, and allylamines) and these largely exert their effects through disruption or inhibition of peripheral cell structures, including the plasma membrane or cell walls of fungi. Antifungal resistance to all of these agents has been detected, although in the case of polyenes, which disrupt the fungal cell membrane through binding to ergosterol, this is of lower prevalence and poorly characterised. Unfortunately, polyene agents have the notable disadvantage of having the highest host cell toxicity profiles and being poorly adsorbed, which limits their application. Azole antifungals inhibit synthesis of the cell membrane by interfering with the cytochrome P450-dependent enzyme lanosterol 14-alpha-demethylase. This enzyme converts lanosterol to ergosterol, which is the principal sterol in fungal cell membranes. Mutations in the *Candida ERG11* gene (Whaley et al., 2017), which encodes lanosterol demethylase, can lead to azole resistance. Echinocandins inhibit glucan synthesis in the cell wall of *Candida* and mutations of the *FKS1* gene, which produces the β-D-1,3-glucan synthase complex, which is frequently involved in resistance (Dudiuk et al., 2015). Other mechanisms of resistance to antifungals exist, and include the overexpression of efflux pumps, the use of surrogate or external sources of sterols, and also the growth of *Candida* within biofilms. Biofilms are, indeed, the main form of growth through which *Candida* colonises both abiotic and biotic surfaces in the human body. Importantly, these *Candida* biofilms exhibit an inherently higher tolerance to antifungal agents (Pereira et al., 2021).

Therefore, there is a clear need for the development of alternative antifungal agents, and new agents should preferably have antibiofilm activity and a low propensity for generating microbial resistance. This article reports on the further study of a new class of antimicrobial drugs as potential antifungal candidates, XF drugs (Pereira Gonzales and Maisch, 2010; Gonzales et al., 2013). The studied XF drugs (XF-73, XF-70, and DPD-207) are synthetic porphyrins that have intrinsic antibacterial effects through binding and disrupting bacterial cell membranes (Ooi et al., 2009). As XF-73 and XF-70 contain a porphyrin ring within their structure, this facilitates a second, light-activated antibacterial mechanism of action, known as photodynamic therapy (PDT), whereby XF-73 and XF-70 release reactive oxygen species when activated by light at a wavelength of 420 nm (Maisch et al., 2005), which can enhance antibacterial activity. By design, DPD-207 does not exhibit PDT activity. Although several investigations have assessed the antibacterial effects of XF drugs (Farrell et al., 2010; Board-Davies et al., 2022), studies into their antifungal activity remain comparatively limited. XF-73 has recently completed a Phase 2 clinical trial demonstrating significant antibacterial activity against intranasal carriage of *Staphylococcus aureus* in patients, (Mangino et al., 2023), and, therefore, clinical safety data for this compound are available for multiple, topical dosing in the mucous membrane, opening a potential pathway for the development as a new antifungal treatment.

The aim of this research was, therefore, to undertake the *in vitro* evaluation of XF drugs for activity against six strains of the fungal pathogen *C. albicans* in both planktonic and biofilm cultures. The effect of treatment on reducing the damage caused by the *C. albicans* SC5314 biofilm infection of an *in vitro* mucosal epithelial model was also assessed. It was envisaged that successful demonstration of antifungal activity would contribute to expanding the repertoire of candidate antifungal therapies.

## Materials and methods

### Assessment of anti-candidal activity of XF drugs

Several experimental approaches were used to assess the antifungal activity of the XF drugs. These included minimum inhibitory concentration (MIC) measurements using a modified broth microdilution method, the determination of minimum biofilm eradication concentrations (MBECs), the live/dead staining of biofilms, and the assessment of *C. albicans* biofilm-induced damage to a reconstituted human oral epithelium (RHOE).

### Preparation of *Candida* species and strains

The strains of *C. albicans* (*n* = 6) (**Table 1**) used to assess the antifungal effects of the XF drugs were maintained by culture on Sabouraud dextrose agar (SDA) at 37°C until required for the experiments. Apart from *C. albicans* SC5314, all test strains originated from the oral cavity. One strain (*C. albicans* PB1) was derived from a healthy oral mucosa, the remaining strains were from patients attending the School of Dentistry, Cardiff University, with oral candidosis. The strains were, therefore, representative of a range of oral conditions and pathologies (Malic et al., 2007). All incubations unless otherwise stated were under stationary conditions.

**Table 1:** Minimum inhibitory concentrations of XF drugs against strains of *Candida albicans*.

Candida strain	Minimum inhibitory concentrations (µg/mL) of XF-drugs				
	XF-73	XF-73 with PDT	XF-70	XF-70 with PDT	DPD-207
***C. albicans* 480/00**	0.5	0.5	2	1	8
***C. albicans* PB1/93**	0.5	0.5	2	0.5	8
***C. albicans* 480/99**	0.5	0.25	2	0.25	16
***C. albicans* PTR/93**	0.25	0.5	0.5	0.5	8
***C. albicans* 109/93**	0.5	0.25	0.25	0.25	4
***C. albicans* SC5314**	0.5	0.5	1	1	8

The MICs represent the mode of the results from the triplicate broth microdilution assays. No minimum biofilm eradication concentration values were reported at concentrations up to 1,024 µg/mL. Photodynamic therapy (PDT).

### Preparation of XF drugs

Three XF drugs (XF-73, XF-70, and DPD-207; Destiny Pharma plc) were resuspended in distilled water to generate stock concentrations of 10 mg/mL. These preparations were stored for up to 1 week at 4°C prior to use in these studies.

### Minimum inhibitory concentration of XF drugs against *Candida albicans*

Broth microdilution was used to measure the minimum inhibitory concentration (MIC) of each XF drug against planktonic cultures of six strains of *C. albicans*. The method was based on the broth microdilution method of the Clinical and Laboratory Standards Institute (CLSI, formerly NCCLS) (The National Committee for Clinical and Laboratory Standards, 2002). Briefly, 100 μL of the XF drug (XF-73, XF-70, and DPD-207) at concentrations between 0 μg/mL and 512 μg/mL in Roswell Park Memorial Institute (RPMI) 1640 medium was added to the wells of 96-well microtitre plates. The cultures of *C. albicans* in RPMI 1640 medium were adjusted to a 0.5 McFarland standard and further diluted 10-fold. A 5-μL volume of these cultures was added to the wells containing the different drug concentrations. For selected plates containing XF-73 and XF-70, the effect of 15 minutes of PDT was also assessed by illumination using a modified light source (Waldmann Medizintechnik, Villingen-Schwenningen, Germany; at wavelengths ranging from 380 nm to 480 nm, with peak output at 420 nm) delivering 14 J/cm^2^ of light. Controls included the absence of the drugs (growth control) and also the absence of *C. albicans* (sterility control). All the plates were incubated aerobically at 37°C for 16–20 hours and visually analysed for the growth of *Candida*. The MICs were defined as the lowest concentrations that inhibited *Candida* growth when compared with the drug-free controls. All the tests were performed in triplicate.

### Determination of the minimum biofilm eradication concentration

The XF drug treatment of *C. albicans* biofilms was tested based on a minimum biofilm eradication concentration (MBEC) assay (Board-Davies et al., 2022). Briefly, the cultures of all six strains of *C. albicans* were grown in Sabouraud dextrose broth (SDB) overnight at 37°C. The broth cultures were then diluted in SDB to an optical density (OD) at 620 nm of 1.0 (equivalent to 1–5 × 10^6^ cells/mL). This preparation was then diluted 20-fold into the wells of a microtitre plate containing SDB. The plates were incubated for 24 hours at 37°C to facilitate biofilm formation. Planktonic *Candida* was then removed using a pipette and the biofilm was washed with 100 µL of phosphate-buffered saline (PBS). Following this, the XF drugs were added in a twofold dilution series (final concentration range 0.5–1024 µg/mL in SDB) to the appropriate wells. The control wells were those with no drug added or an absence of *C. albicans*. Selected plates for PDT were placed on a light box for 15 minutes, as described above. Microtitre plates were then incubated at 37°C for 24 hours. The medium was removed and the biofilm in the wells was washed (× 2) with PBS. The biofilm was then resuspended by repeat pipetting in 100 µL of SDB and the OD at 620 nm measured. After a further 24-hour incubation at 37°C, the OD at 620 nm was again measured and the differences in the values used to establish biofilm regrowth. The changes in turbidity were initially assessed visually and MBECs were subsequently defined as the XF drug concentrations where a < 20% change in the OD at 620 nm occurred between the two measurements. All the experiments were conducted in triplicate.

### Assessment of antifungal effects of XF drugs by confocal laser scanning microscopy

*Candida albicans* SC5314 biofilm was grown on polycarbonate coupon surfaces in a CDC biofilm reactor (Biosurface Technologies, Bozeman, MT, USA). *Candida albicans* SC5314 was selected for use, as it is considered a reference strain (Bartelli et al., 2018), and was confirmed to be an adept biofilm-forming isolate from the MBEC determination work. Briefly, *C. albicans* was cultured overnight at 37°C in SDB and the cell concentration standardised by adjusting to an OD at 620 nm of 1.0 with fresh culture medium. A 4-mL volume of this *C. albicans* inoculum was then added to a bioreactor containing SDB, which was incubated, with stirring, under batch conditions for 24 hours. After incubation, bioreactor rods holding the polycarbonate coupons were transferred to a new bioreactor and incubated for a further 24 hours under continuous flow conditions. The polycarbonate coupons with biofilms were then removed from the bioreactor and immersed in different concentrations of XF drugs for 24 hours, with selected treatments also incorporating light activation, as described above. The biofilms were then live/dead stained (Live/Dead™ BacLight™ viability kit; Thermo Fisher Scientific, Paisley, UK) and imaged by confocal laser scanning microscopy (CLSM) to assess the biocidal effects of the XF drugs.

### Effect of XF drug treatment on *Candida albicans* biofilm infection of a reconstituted human oral epithelium

For these studies, only *C. albicans* SC5314 was employed due to the limited availability of reconstituted human oral epithelium. The *Candida albicans* SC5314 biofilm was generated using the CDC bioreactor, as described above. The biofilm was then treated with the XF drugs (1,024 µg/mL ± light activation) and overlaid on a reconstituted human oral epithelium (RHOE; Skin Ethic Laboratories), which was incubated for 16 hours at 37°C. The RHOE specimens were removed from the maintenance medium and fixed overnight in 10% formal saline solution. The tissues were then subjected to dehydration and paraffin wax embedding in pathology cassettes using a Leica ASP300S processor. The tissue sections (5 µm thick) were obtained from the rehydrated tissues and stained using a *C. albicans* peptide nucleic acid (PNA) probe (Yeast Traffic Light® PNA FISH^®^ kit; AdvanDx, Vedbæk, Denmark) and a nucleic acid stain, as described in previous studies (Silva et al., 2011; Cavalcanti et al., 2015). The levels of lactate dehydrogenase (LDH) in the RHOE maintenance medium were measured using the Invitrogen CyQUANT™ LDH cytotoxicity assay (Fisher Scientific UK Ltd, Loughborough, UK) in accordance with the manufacturer’s recommended protocol. Higher levels of LDH would be indicative of increased tissue damage.

### Statistical analyses

Results for the MIC and MBEC for each isolate are expressed as the modal averages for the replicates for each isolate. One-way ANOVA followed by Tukey’s multiple comparisons test was used to evaluate the statistical differences in LDH levels between samples at 95 % confidence.

## Results

### XF drug antifungal activity against *Candida albicans* using broth microdilution, MBEC and live/dead staining assays

The MICs for XF-73, XF-70 and DPD-207 (± light activation, where appropriate) were determined for six different *C. albicans* strains. **Table 1** summarises the MICs, which, for XF-73 and XF-70, were between 0.25 µg/mL and 2 µg/mL, depending on the *C. albicans* strain. The MICs for DPD-207 were higher and ranged between 4 µg/mL and 16 µg/mL. Light activation did not impact MICs for XF-73 or XF-70, which ranged between 0.25 µg/mL and 1 µg/mL with PDT.

The effectiveness of XF drugs against the biofilms of the six *C. albicans* strains was also examined using the MBEC assay. However, no MBECs were measurable (up to the maximum concentration tested of 1,024 µg/mL) in this microtitre plate-based assay.

**Figure 1:**
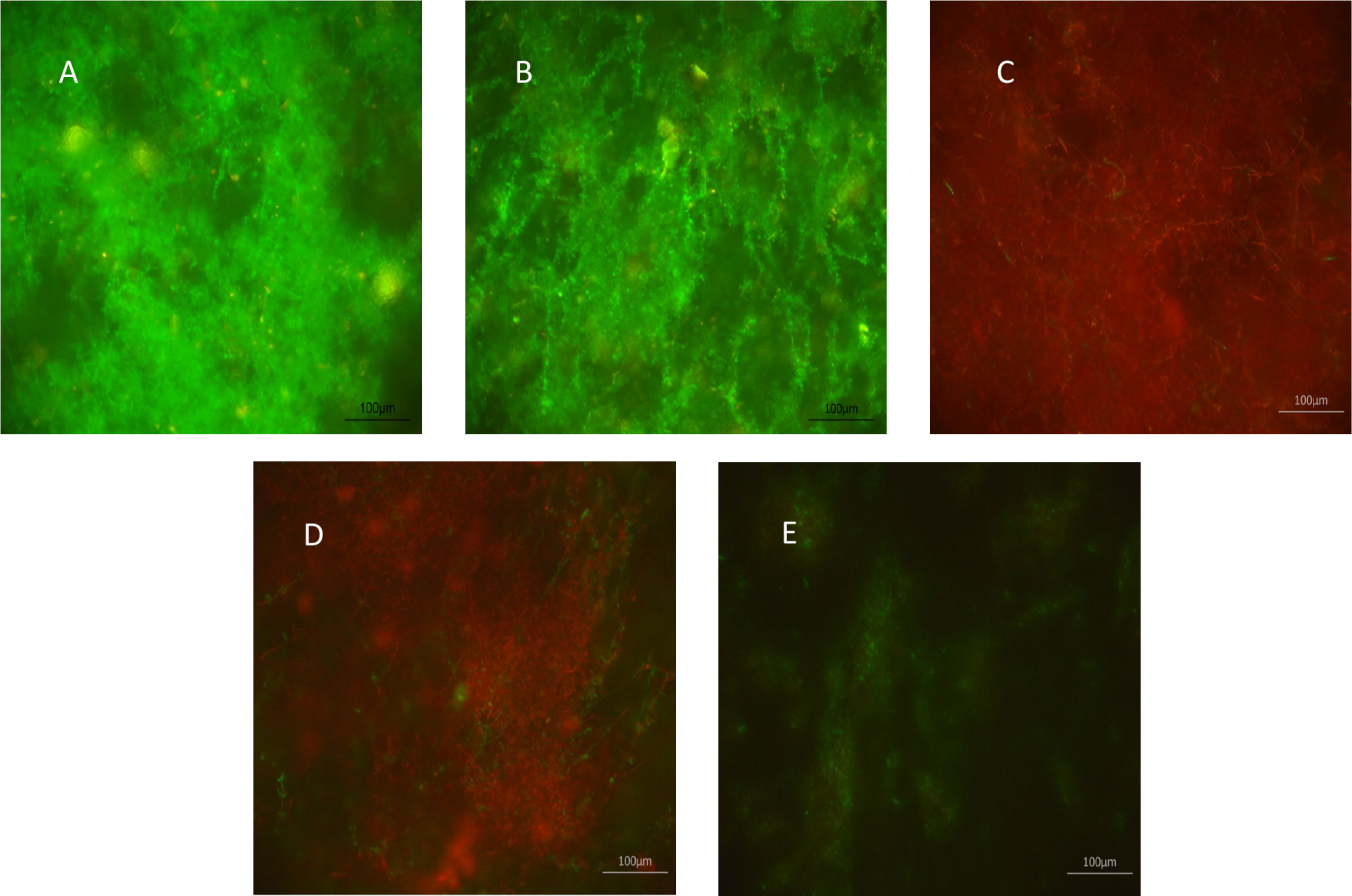
Effect of XF-drug treatment on *Candida albicans* SC5314 biofilms. **(A)** Untreated control; **(B)** light alone; **(C)** XF-73 (64 µg/mL); **(D)** XF-70 (64 µg/mL); and **(E)** DPD-207 (256 µg/mL). Green SYTO™ 9 staining represents live *C albicans* cells. Red propidium iodide staining represents dead *C albicans* cells.

The effects of XF drugs on a *C. albicans* SC5314 biofilm attached to polycarbonate coupons were also assessed by live/dead staining and CLSM. **Figure 1** shows that XF-73 and XF-70 resulted in a significant killing of *C. albicans* SC5314 biofilm cells at a concentration of 64 µg/mL, as seen by the predominance of red (propidium iodide) staining (images C and D), irrespective of inclusion of light activation. DPD-207 reduced the number of viable *C. albicans* SC5314 biofilm cells at a concentration of 256 µg/mL.

### Effect of XF drug treatment of *Candida albicans* biofilm on pathogenicity

**Figure 2:**
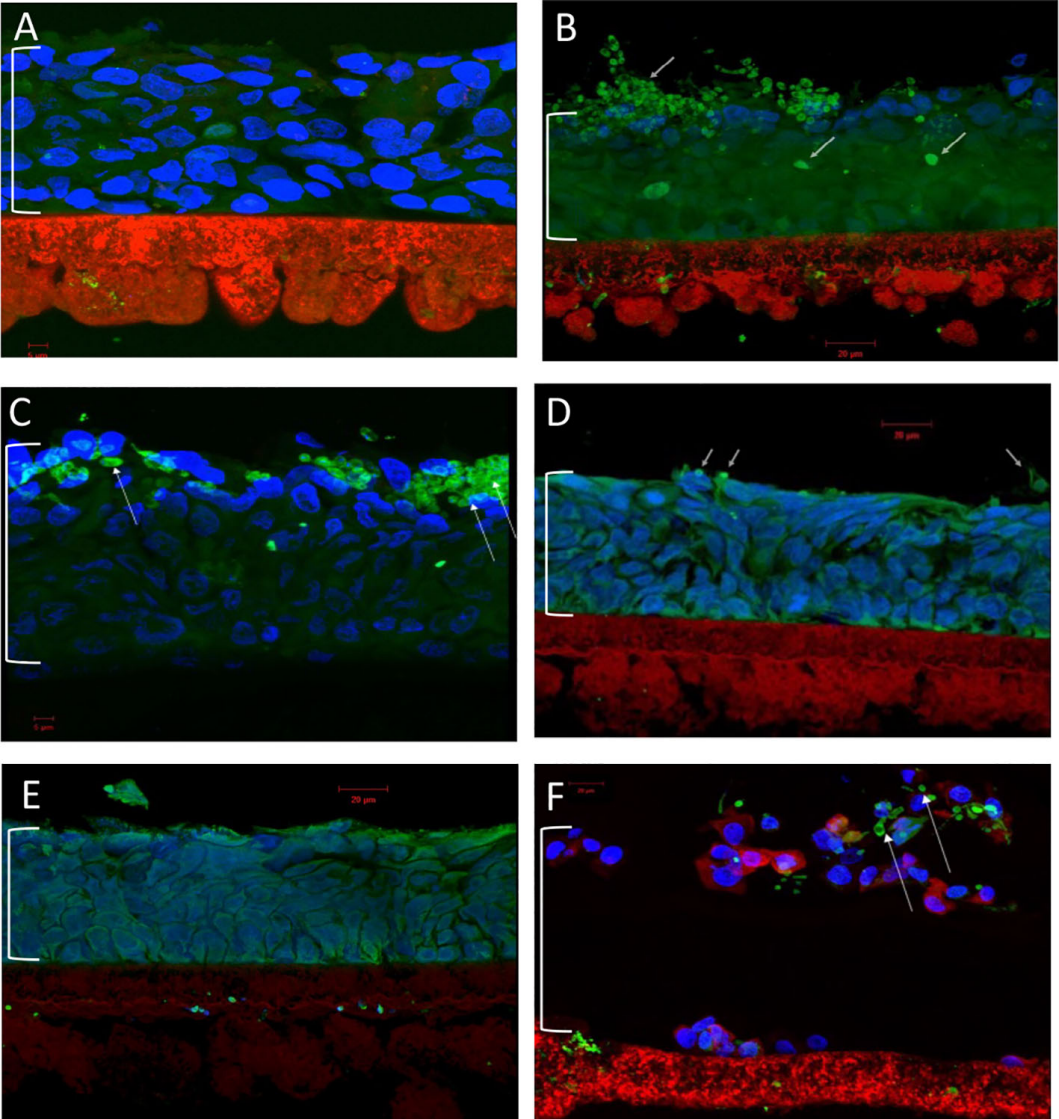
Confocal laser scanning microscopy of reconstituted human oral epithelium infected with *Candida albicans* SC5314 biofilms. **(A)** Non-infected control; **(B)** infected (untreated); **(C)** infected (light alone); **(D)** infected (XF-73 treated); **(E)** infected (XF-70 treated); and **(F)** infected (DPD-207 treated). Blue, epithelial cell nuclei; Green and arrowed, *Candida albicans* SC5314 cells. The bracket indicates the epithelium layer.

**Figure 3:**
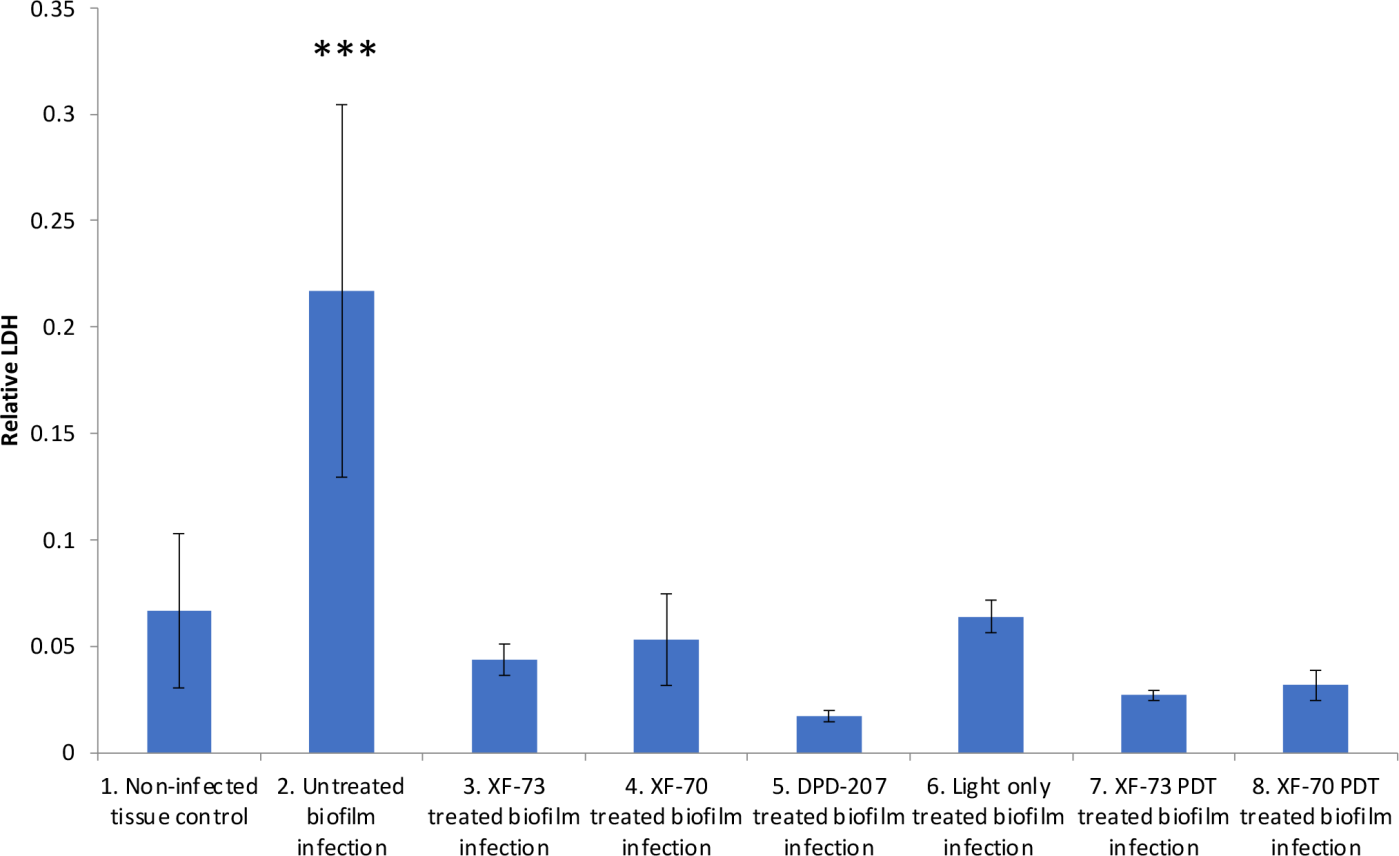
Comparative lactate dehydrogenase (LDH) activity in conditioning medium of reconstituted human oral epithelium infected with *Candida albicans* SC5314 biofilms treated with XF-73, XF-70 (1024 µg/mL with or without light activation) and DPD-207 (1,024 µg/mL). LDH activity was the 680nm absorbance value (background) subtracted from the 480nm absorbance value The bars show averages and SDs within each group. *** denotes significance at a *p*-value < 0.005. (One-way ANOVA followed by Tukey’s multiple comparisons test).

The final antifungal assessment examined the effects of XF drug-treated and untreated *C. albicans* SC5314 biofilms on the subsequent infection of a RHOE. **Figure 2** presents the CLSM images of the RHOE following exposure to biofilms previously exposed to different XF drug treatment types. Both XF-73 and XF-70 reduced *C. albicans* SC5314 invasion of the RHOE. In most cases, no *Candida* cells were detected in the tissue exposed to treated biofilms, irrespective of inclusion of light activation. **Figure 3** presents the relative damage to the RHOE tissue based on measuring LDH activity in the conditioning medium occurring as a response to biofilm infection. XF-70, XF-73, and DPD-207 all significantly reduced tissue damage when compared with untreated biofilm infected tissue (positive control). No further reduction of tissue damage was observed when light activation was also used in the presence of the XF drugs, although the biofilm exposed to light activation alone showed lowered LDH levels, which was unexpected given the absence of a biocidal effect on *C. albicans* from light activation only, which was based on live/dead staining (**Figure 1**).

## Discussion

Given the clear need for new effective antifungals, which are also effective in combating biofilms containing *Candida*, this study aimed to determine the effectiveness of three antibacterial XF drugs in inhibiting strains and species of *Candida*. Six strains of *C. albicans* were used in the study and the effects against planktonic and biofilm growth were determined.

Initially, the effectiveness of the XF drugs (XF-73, XF-70, and DPD-207) was assessed by determining both the MICs and MBECs of each drug (± light activation, where appropriate). The innate anti-*Candida* activity of XF drugs was confirmed for all of the agents tested. Both XF-73 and XF-70 have a non-metalated porphyrin ring within their structures, which on light activation leads to the release of reactive oxygen species and may provide a second mechanism of anti-candidal action. However, when light activation was tested, no enhanced antifungal effect was observed. Gonzales et al. (2013) previously showed enhanced fungicidal PDT effects with XF-73 against a single planktonic strain of *C. albicans* (ATCC-MYA-273) and its biofilm. This previous research was based on immediately quantifying colony-forming units (CFUs) post PDT; a six-log reduction of viable planktonic CFUs was reported but, importantly, not the complete killing of all the fungal cells. The methodology was, therefore, different to the MIC methodology used in the present study, which would have allowed the surviving cells to regrow. In this study, the failure to record an MBEC value was not indicative of an absence of antibiofilm activity, as any viable cells that persisted after treatment would lead to subsequent regrowth (even if the majority of biofilm cells had been killed). Indeed, although Gonzales et al. (2013) did not report MBEC values at the tested concentrations, they did report a five-log reduction of *C. albicans* biofilm cells using PDT with XF-73, although their approach employed an immediate assessment of effects without the opportunity of biofilm regrowth. As a consequence, antibiofilm effects were further examined in this study. In these experiments, the *C. albicans* SC5314 biofilm on polycarbonate coupons was treated with each XF drug (up to a concentration of 1,024 µg/mL) and the effects analysed by live/dead staining. In these assays, it was clear that all of the XF drugs possessed significant *Candida* antibiofilm activity.

Previously, research has used the RHOE to compare *Candida* virulence (Silva et al., 2011), investigate immune responses (Schaller et al., 2002), and evaluate antifungal effects (Boros-Majewska et al., 2014) based on subsequent tissue damage. In this study, a biofilm of *C. albicans* SC5314 on polycarbonate coupons was produced and treated with XF drugs (with and without light activation). Importantly, the XF drug-treated *Candida* biofilm caused significantly reduced damage to the RHOE than the untreated infected controls, confirming the potential of these agents to have a clinical impact. Interestingly, light alone also reduced the subsequent LDH activity of infection, which was unexpected as no anti-candidal biofilm effect from light (without XF drug treatment) had been previously observed. Indeed, Gonzales et al. (2013) reported that light alone did not kill *C. albicans* based on the measurements of cell growth. Further investigations are, therefore, warranted to establish the basis of the reduction of LDH activity with light alone observed in this study, but it might be indicative of some detrimental effect on *C. albicans* biofilms that was not highlighted by live/dead staining. A favourable clinical safety profile at higher concentrations of XF-73 than was tested within the RHOE tissue model has previously been reported (Mangino et al., 2023), but the efficacy and safety profile of XF-73 will need to be further investigated using suitable *in vivo* models, such as the murine model described by Segal and Frenkel (2018).

## Conclusions

This research demonstrated that XF drugs were effective antifungal agents against *C. albicans* and its biofilms via the drugs’ innate antimicrobial mechanism of action. XF-73 and XF-70 were the most potent antifungal agents tested, whereas DPD-207 required higher drug concentrations for an antifungal effect. The induction of the secondary PDT mechanism of action via light activation did not enhance antifungal activity against planktonic or biofilm cultures with XF-73 and XF-70. The low MIC values reported, coupled with antibiofilm effects leading to protection from tissue damage, would suggest that XF drugs can be considered as potential treatment alternatives for superficial candidoses. Further work is warranted to assess XF drug activity against other *Candida* species and strains with known resistance to traditional antifungals and to optimise delivery mechanisms to facilitate the translation to clinical use on mucosal membranes.

## Data availability statement

The raw data supporting the conclusions of this article will be made available by the authors, without undue reservation.

## Author contributions

DW and EB-D conceived the experiments. EB-D performed the experiments. EB-D, DW, WL, DH, and WR-W reviewed the data. DW and EB-D wrote the manuscript. All authors contributed to the article and approved the submitted version.
